# Targeting heat-shock protein 90 with ganetespib for molecularly targeted therapy of gastric cancer

**DOI:** 10.1038/cddis.2014.555

**Published:** 2015-01-15

**Authors:** H Liu, J Lu, Y Hua, P Zhang, Z Liang, L Ruan, C Lian, H Shi, K Chen, Z Tu

**Affiliations:** 1 School of Pharmacy, Jiangsu University, Zhenjiang, Jiangsu 212013, China; 2 School of Medicine, Jiangsu University, Zhenjiang, Jiangsu 212013, China; 3 Institute of Oncology, Affiliated Hospital of Jiangsu University, Zhenjiang, Jiangsu 212013, China; 4 Institute of Life Sciences, Jiangsu University, Zhenjiang, Jiangsu 212013, China

## Abstract

Gastric cancer (GC) remains the fifth most common cancer worldwide. Heat-shock protein 90 (HSP90) has become an attractive therapeutic target in treating cancers, because of its abnormally high expression in cancers. Several successful cases of HSP90 inhibitors capable of inhibiting GC inspired us to try ganetespib, a clinically promising and actively investigated second-generation HSP90 inhibitor in GC treatment. In our study, we show that ganetespib markedly reduced the growth of MGC-803 and also significantly inhibited the growth of SGC-7901 and MKN-28 in a dose-dependent manner. It induced G2/M cell-cycle arrest and apoptosis in all three cell lines, together with the related markers affected significantly. Mechanistically, ganetespib caused pronounced decrease of expression of classic HSP90 client proteins. Specifically, it greatly affected epidermal growth factor receptor (EGFR) signaling cascades by markedly decreasing the levels of total EGFR and EGFR on cell membranes. EGFR knockdown also induced cell-cycle arrest and apoptosis accompanied with a decrease of several EGFR downstream proteins. These results strongly support that EGFR signaling greatly contributes to the ganetespib inhibitory effects. Besides, we found that the responses of GC cell lines to ganetespib correlated well with their EGFR expression levels: MGC-803, as well as AGS and BGC-803, with higher EGFR expression responded to ganetespib better, whereas SGC-7901 and MKN-28 with lower EGFR levels were much less sensitive to ganetespib. Although SGC-7901 and MKN-28 were not very sensitive to ganetespib, ganetespib worked synergistically with radiation and cisplatin in killing them. Importantly, ganetespib significantly inhibited the growth of xenograft tumors *in vivo* as a single agent or in combination with cisplatin. Results of hematoxylin/eosin staining, TUNEL (terminal deoxynucleotidyl transferase dUTP nick-end labeling) assays, and immunohistochemistry staining of phosphorylated cyclin-dependent kinase 1 (pCDK1), EGFR and Ki-67 revealed significant differences in ganetespib-treated tumors. Collectively, our data suggest that ganetespib, as a new potent treatment option, can be used for the molecularly targeted therapy of GC patients according to their expression profiles of EGFR.

Gastric cancer (GC) remains the fifth most common cancer worldwide, with an estimated 9 52 000 new cases (7% of total cancer incidence) and 7 23 000 deaths (9% of total cancer mortality) in 2012.^1^ As a highly aggressive and lethal malignancy, the aggressive nature of GC is linked to mutations in tumor suppressor genes, oncogenes, growth factors and their receptors, and so on.^2^ Till now, there are few effective treatment options for advanced patients with distant metastasis or recurrence.^3^ The detailed mechanisms that regulate GC are not yet fully understood; therefore, such situations underscore the persistent unmet need to identify therapeutics that target pathways involved in GC progression.

Consequently, identification of key regulatory molecules in GC is of high priority for understanding the mechanism for tumor dissemination as well as the development of novel interventions. Aberrant expression and kinase activity of Src have been found in many different tumors, including GC.^4, 5^ Previous studies have shown that phosphorylated mammalian target of rapamycin (p-mTOR) was significantly overexpressed in advanced GC patients' tumors and suggested that the PI3K/AKT/mTOR (phosphoinositide 3-kinase/AKT/mTOR) pathway is activated in GC with potential prognostic and predictive significance.^6, 7^ Aurora A overexpression has recently been reported in GC, and it was suggested to be associated with cancer progression and poor prognosis.^8, 9, 10^

In our previous work, we conducted data mining meta-analyses integrating results from multiple small interfering RNA (siRNA) screens to identify gene targets, which are necessary for the growth of different cancer cells. Among those genes, we found that heat-shock protein 90 (HSP90) was one of the most vital proteins for cancer cell survival.^11^ As we know, HSP90 is involved in the regulation of numerous proteins important for GC pathogenesis, such as proteins important for cell adhesion (e.g., focal adhesion kinase), cell motility (e.g., epidermal growth factor receptor (EGFR), c-Src, phosphoinositide-dependent protein kinase 1 (PDK1)), and angiogenesis (e.g., hypoxia-inducible factor-1 (HIF-1), vascular endothelial growth factor receptor (VEGFR)).^12, 13, 14, 15^ For these reasons, HSP90 has been of considerable interest as a therapeutic target in GC.

As an ATP-dependent molecular chaperone protein, HSP90 conducts the proper folding of myriad proteins.^12, 14^ Abnormally high expression of HSP90 has been found in GC and been greatly considered as an independent prognostic marker of GC progression.^16, 17, 18^ HSP90 remains an attractive therapeutic target in a variety of cancers,^19, 20, 21, 22^ and inhibition of HSP90 showed potent growth inhibitory effects on GC in cell cultures and in mouse models.^23, 24, 25^ Ganetespib is a particularly promising second-generation HSP90 inhibitor that does not suffer from the toxicity issues associated with earlier-generation HSP90 inhibitors and exhibits increased potency compared with first- and other second-generation agents.^11, 26, 27, 28, 29^

In this current study, using cell culture and xenograft mouse models, we sought to evaluate the effects of ganetespib treatments on GC cells, individually or in combination with other treatments. In addition, we searched for the possible mechanisms underlying the antitumor activities of ganetespib. And, our results suggested that, as a promising drug candidate, ganetespib has potent antitumor activities on GC, and it is worth being investigated further clinically for the molecularly targeted therapy of GC patients.

## Results

### Ganetespib treatment inhibits viabilities of GC cell lines

**Figure 1:**
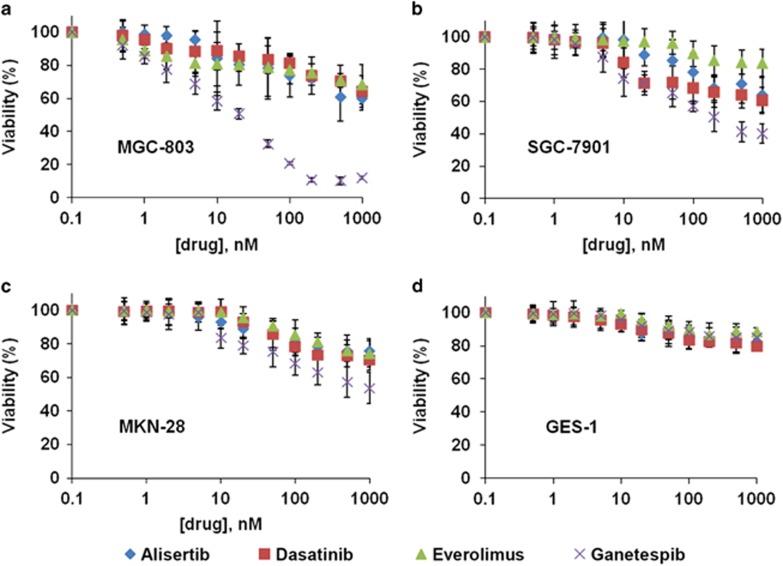
Ganetespib treatment inhibits viabilities of GC cell lines. Cells of GC cell lines MGC-803 (**a**), SGC-7901 (**b**), MKN-28 (**c**), and normal gastric mucosal epithelial cell line GES-1 (**d**) were treated with increasing concentrations of dasatinib (red squares), everolimus (green triangles), alisertib (blue diamonds), and ganetespib (purple crosses) for 72 h and cell viability was assessed by MTT assay. Data indicate the mean percentage viability calculated from triplicate samples from multiple independent experiments (*n*≥3) (±S.E.)

On the basis of the connections of GC to Src, mTOR, Aurora A, and HSP90, their corresponding clinically promising inhibitors, dasatinib, everolimus, alisertib, and ganetespib,^11, 28^ were assessed in GC cell lines, including MGC-803, SGC-7901, and MKN-28 cells, and normal gastric mucosal epithelial cell line GES-1. Unexpectedly, the three drugs dasatinib, everolimus, and alisertib only showed limited GC killing efficacy, and cells remained 50–90% viable even at high drug concentrations (Figures 1a–d and data not shown). However, ganetespib treatment resulted in a dose-dependent inhibition of cell viabilities in GC cell lines. The half-maximal inhibitory concentration (IC_50_) value of MGC-803 cells is 16.7 nM, whereas the IC_50_ values of SGC-7901 and MKN-28 cell lines are about 171.9 and 582 nM (Figures 1a–c), respectively. More importantly, GES-1 cells tolerated ganetespib very well as they maintained about 85% viability when the drug concentration reached 1000 nM. These results indicated that with highly potent anticancer activity, ganetespib has greater specificity to GC cells, rather than to normal gastric mucosal epithelial cells (Figure 1d).

### Short-term treatment of ganetespib induces G2/M cell-cycle arrest in GC cells

**Figure 2:**
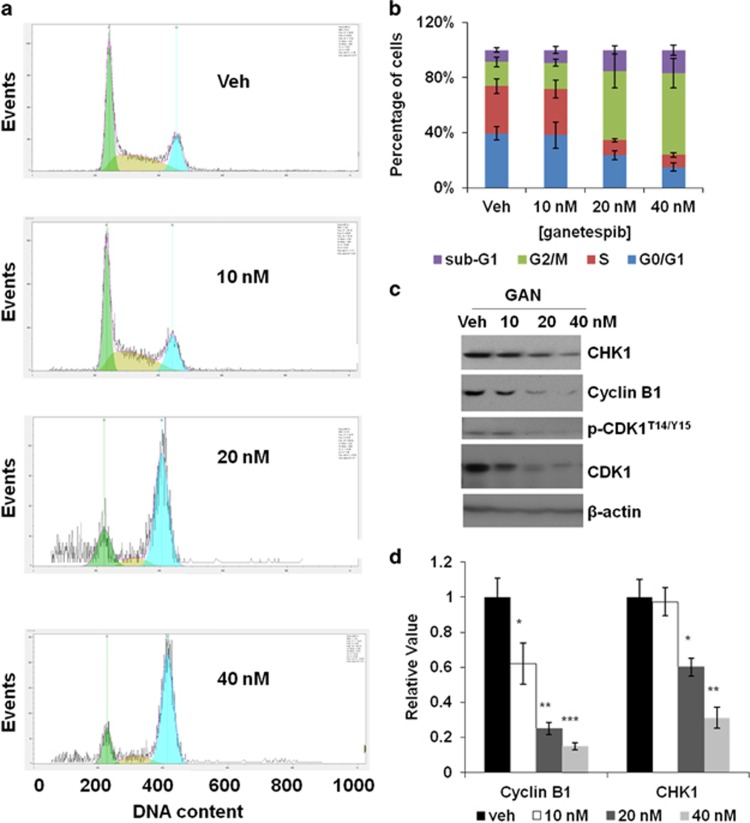
Short-term ganetespib treatment induces G2/M cell-cycle arrest in GC cells. (**a**) Representative flow cytometry plots for MGC-803 cells. Cells were treated with 0, 10, 20, or 40 nmol/l ganetespib for 24 h, stained with propidium iodide and analyzed for cell-cycle distribution. (**b**) Data shown are the mean values (±S.E.) of each cell-cycle phase from three independent experiments. (**c**) MGC-803 cells were treated with increasing doses of ganetespib for 24 h and protein lysates were subjected to immunoblot analysis with the indicated antibodies of G2/M cell-cycle markers. (**d**) Detection of cyclin B1 and CHK1 levels present in protein lysates by ELISA assay. Data indicate the mean relative values calculated from three independent experiments (±S.E.). Statistically significant differences with *P*<0.05 were considered significant (*P<0.05; **P<0.01; ***P<0.001)

As we found that ganetespib greatly inhibited the growth of GC cells, we were interested in finding out how ganetespib affected cell growth. To do that, cell-cycle distribution of MGC-803 cells after ganetespib treatment was analyzed. As shown in Figure 2a, a 24-h exposure of MGC-803 cells to ganetespib (20 and 40 nM) resulted in a marked increase of G2/M-phase cells, which was accompanied by a decrease in G1/G0- and S-phase cells (Figure 2b). At the same time, average percentage of cells in the sub-G1 phase also increased from 8.7% (in the vehicle group) to 16.8% (treated with 40 nM ganetespib). Similarly, exposure to ganetespib in SGC-7901 cells also resulted in the accumulation of cells in the G2/M phase just at higher concentrations of ganetespib (160 and 320 nM), which was consistent with its higher IC_50_ value (Supplementary Figure 1a). As expected, in MKN-28 cells the percentage of the G2/M-phase cells did not increase as much as that in MGC-803 cells even when treated with ganetespib at 320 nM, which is consistent with its much higher value of IC_50_ (Supplementary Figure 1b). Expression of CHK1 (checkpoint kinase 1), cyclin B1, phospho-CDK1^T14/Y15^, and CDK1 in MGC-803 cells all decreased after they were exposed to ganetespib for 24 h (Figure 2c), demonstrating G2/M cell-cycle arrest. The decrease of the protein levels of cyclin B1 and CHK1 was also confirmed with enzyme-linked immunosorbent assays (ELISA) as shown in Figure 2d.

### Long-term treatment of ganetespib induces apoptosis in GC cells

**Figure 3:**
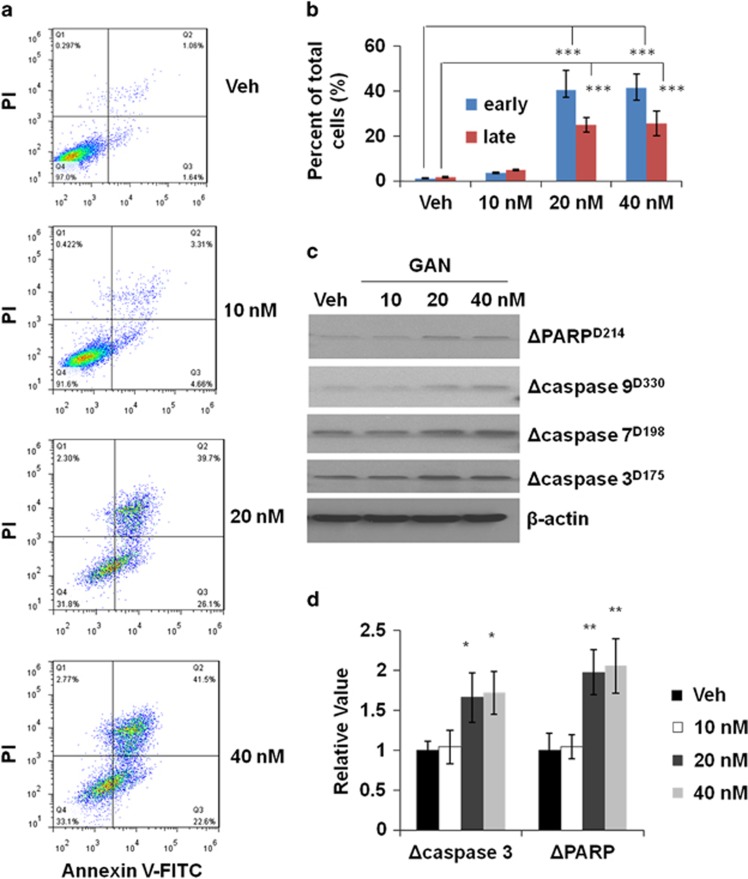
Long-term ganetespib treatment induces apoptosis in GC cells. (**a**) Representative flow cytometry plots for MGC-803 cells. Cells were treated with 0, 10, 20, or 40 nmol/l ganetespib for 72 h, stained with Annexin V-FITC/propidium iodide, and analyzed for cell apoptosis distribution. (**b**) The above experimental results were analyzed for the presence of Annexin V (+)/PI (−) (early apoptosis) and Annexin V (+)/PI (+) (late apoptosis). Data shown are the mean values (±S.E.) from three independent experiments. (**c**) MGC-803 cells were treated with increasing doses of ganetespib for 72 h and protein lysates were subjected to immunoblot analysis with the indicated antibodies of apoptosis markers. (**d**) Detection of cleaved caspase-3 and cleaved PARP levels present in protein lysates by ELISA assay. Data indicate the mean relative values calculated from three independent experiments (±S.E.). Statistically significant differences with *P*<0.05 were considered significant (*P<0.05; **P<0.01; ***P<0.001)

As in the above experiment, a significant increase of the sub-G1 cell population was observed after a short-term treatment of ganetespib, we reasoned that apoptosis of cells may increase after the cells were treated with ganetespib for a longer time. Therefore, we treated MGC-803 cells with ganetespib for 72 h. The results showed that there is a significant increase in the percentages of apoptotic cells in both early and late apoptosis stages (Figures 3a and b) upon 20 and 40 nM of ganetespib treatment. Similarly, significant increase in the percentage of apoptotic cells in both early and late apoptosis was also observed in SGC-7901 cells (treated with 160 and 320 nM of ganetespib) (Supplementary Figure 1c). While in the cell line of MKN-28, only 320 nM of ganetespib in the tested range of ganetespib caused an increase of apoptotic cells in early and late stages (Supplementary Figure 1d). The expression of apoptosis markers, cleaved poly ADP ribose polymerase (PARP), and cleaved caspase-3, -7, and -9 all increased depending on the ganetespib dosages in the cell line of MGC-803 (Figure 3c). Furthermore, the increase of expression of cleaved caspase-3 and cleaved PARP were confirmed by the ELISA experiments (Figure 3d). All these markers of cell apoptosis again proved that long-term ganetespib treatment induces cell death through apoptosis.

### Effects of ganetespib on EGFR signaling cascades in GC cells

**Figure 4:**
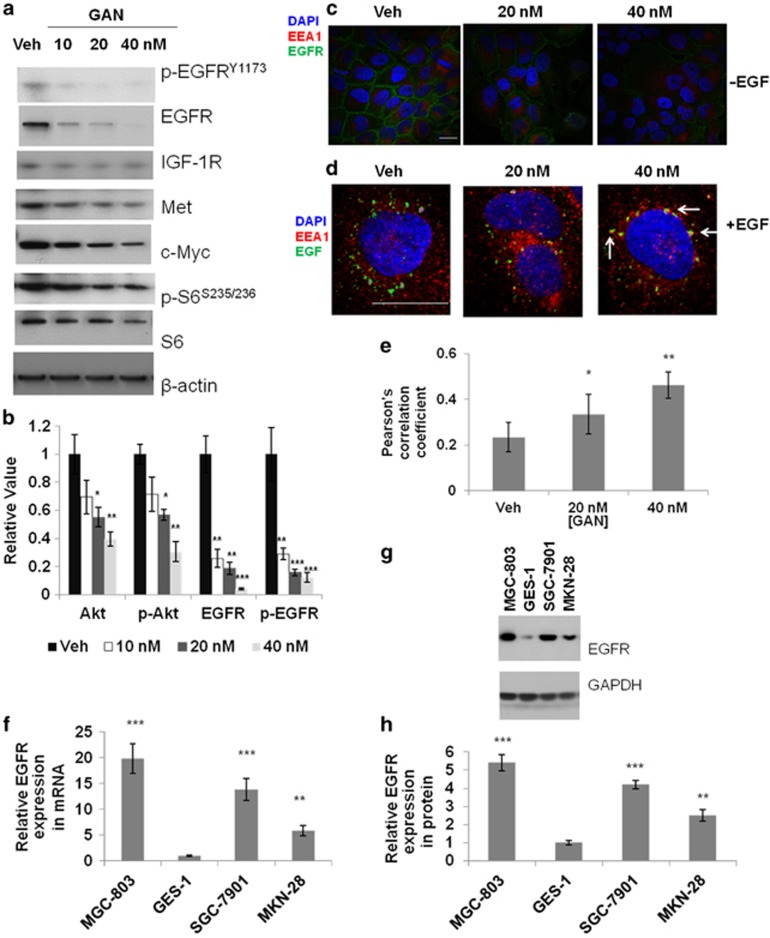
Ganetespib (GAN) treatment affects EGFR signaling cascades in GC cells. (**a**) Ganetespib treatment affects HSP90 client proteins in GC cells. GC cells (MGC-803) were treated with increasing doses of ganetespib for 24 h and cell lysates were subjected to immunoblot analysis with the indicated antibodies. (**b**) Detection of Akt, phospho-Akt^S473^, EGFR, and phospho-EGFR^Y1173^ average levels present in protein lysates by ELISA assay. Data indicate the mean relative values calculated from three independent experiments (±S.E.). (**c**) Ganetespib treatment decreased EGFR (green) on the cell membrane in MGC-803 cells. Scale bar, 5 μm. (**d**) EGFR trafficking was significantly affected by ganetespib pretreatment upon stimulation with fluorescence-labeled EGF (green). Ganetespib treatment caused increased association of internalized EGFR (green) with EEA1-positive early endosomes (red) in MGC-803 cells. Scale bar, 5 μm. (**e**) Quantification of colocalization results from three independent experiment **D**. Pearson's correlation coefficients were analyzed by Image-Pro software (±S.E.). (**f**) Cell lines MGC-803, GES-1, SGC-7901, and MKN-28 were subjected to real-time PCR detection of *EGFR* mRNA. *GAPDH* gene was used as an internal control. Data indicate the mean relative mRNA expression levels from triplicate samples from three independent experiments (±S.E.). (**g**) Representative immunoblots from cell lines MGC-803, GES-1, SGC-7901, and MKN-28 showing EGFR protein expression, with GAPDH as an internal control. (**h**) The mean protein expression levels from three independent experiment **G** (±S.E.). Statistically significant differences with *P*<0.05 were considered significant (*P<0.05; **P<0.01; ***P<0.001)

In addition to the findings that ganetespib can affect GC cell growth by inducing cell-cycle arrest and apoptosis, we are also interested in searching for signaling pathways executing these processes. Therefore, the expression of canonical HSP90 clients, such as phospho-EGFR, total EGFR, IGF-1R (insulin-like growth factor-1 receptor), MET, c-Myc, phospho-S6, and total S6, was measured after 24 h of ganetespib treatment. Results have shown that both phospho-EGFR^Y1173^ and total EGFR were markedly decreased after the cells were treated with ganetespib (Figure 4a), whereas the protein levels of IGF-1R, MET, c-Myc, phospho-S6, and total S6 were only mildly (but significantly) decreased. Similarly, the expression levels of EGFR were also decreased significantly in SGC-7901 cells at 160 nM of ganetespib (Supplementary Figure 1e) and in MKN-28 cells at 320 nM of ganetespib treatment (Supplementary Figure 1f), respectively. Furthermore, the ELISA results not only confirmed the decreasing expression of phospho- and total EGFR but also showed that a downstream effector of EGFR, Akt protein, decreased in total and phosphorylated forms (Figure 4b). In our previous study, EGFR was identified as a top-ranked key regulator in multiple screens and meta-analyses.^11^ Activation of EGFR can affect multiple intracellular oncogenic signaling pathways involved in GC. In addition, EGFR is a classical client protein of HSP90, which is the target of ganetespib in GC cells. All the above information suggested that EGFR-related signaling pathways could greatly contribute to the anticancer activities of ganetespib. To prove this hypothesis, we investigated the effects of HSP90 blockade on functionality and expression of EGFR in cancer cells. Pretreatment of MGC-803 cells with ganetespib for 24 h remarkably decreased the levels of total EGFR (Figures 4a and b) and EGFR constitutively on cell membranes (Figure 4c). Upon stimulation with fluorescence-labeled EGF, EGFR trafficking was also significantly affected by ganetespib pretreatment (Figures 4d and e). Early endosome antigen 1 (EEA1) is the marker for early endosomal compartment in EGFR trafficking. Colocalization of EEA1 and EGFR were greatly increased after ganetespib treatment, with Pearson's correlation coefficients analyzed by Image-Pro software (Media Cybernetics Inc., Warrendale, PA, USA) significantly increased (Figure 4e). We concluded from these results that interference with HSP90 could effectively downregulate EGFR expression, possibly through accelerated EGFR degradation. In addition, we further investigated the mRNA and protein levels of EGFR in four studied cell lines (Figures 4f–h). The results demonstrated that both mRNA and protein levels of EGFR in normal gastric mucosal epithelial cell line GES-1 were markedly lower than those in the GC cell lines. The expression levels of EGFR in cells correlated very well with their responsiveness to ganetespib treatment, as MGC-803, the cell line with highest EGFR expression, has the highest responsiveness to ganetespib (Figure 1), whereas the responsiveness of cell lines SGC-7901 and MKN-28 to ganetespib decreased as their EGFR expression levels decreased. More importantly, these results indicate that ganetespib is especially suitable for treating poorly differentiated GCs, which is the most difficult kind of GC to cure and usually has high EGFR expression.

**Figure 5:**
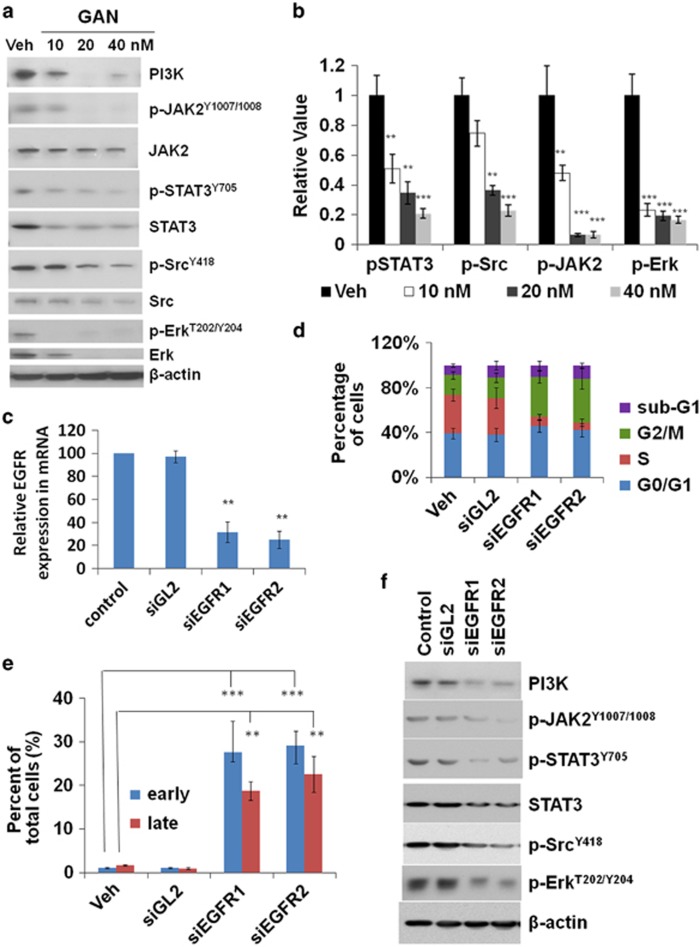
Ganetespib treatment and siRNAs targeting *EGFR* affect EGFR downstream pathways similarly in GC cells. (**a** and **b**) Ganetespib treatment affects EGFR downstream pathways in GC cells. (**a**) MGC-803 cells were treated with increasing doses of ganetespib for 24 h and protein lysates were subjected to immunoblot analysis with the indicated antibodies of EGFR downstream pathway proteins. (**b**) Detection of average levels of phospho-STAT3^Y705^, phospho-Src^Y418^, phospho-JAK2^Y1007/1008^, and phospho-Erk^T202/Y204^ present in cell lysates by ELISA assay. Data indicate the mean relative values calculated from three independent experiments (±S.E.). (**c**–**f**) siRNA knockdown affects EGFR downstream pathways similar to ganetespib treatment in GC cells. (**c**) The cell line MGC-803 was transfected with either vehicle, siGL2, siEGFR1, or siEGFR2, and cells were subjected to real-time PCR detection of *EGFR* mRNA levels. *GAPDH* was used as an internal control. Data indicate the mean relative mRNA expression levels from triplicate samples from three independent experiments (±S.E.). (**d**) Data shown are the mean values (±S.E.) of each cell-cycle phase from three independent experiments. MGC-803 cells were transfected with either vehicle, siGL2, siEGFR1, or siEGFR2 for 24 h, stained with propidium iodide, and analyzed for cell-cycle distribution. (**e**) The percentage of cells stained with Annexin V (+)/PI (−) (early apoptosis) and Annexin V (+)/PI (+) (late apoptosis). Data shown are the mean values (±S.E.) from three independent experiments. MGC-803 cells were transfected with either vehicle, siGL2, siEGFR1, or siEGFR2 for 72 h, stained with Annexin V-FITC/propidium iodide, and analyzed for cell apoptosis distribution. (**f**) MGC-803 cells were transfected with either vehicle, siGL2, siEGFR1, or siEGFR2 for 24 h, and protein lysates were subjected to immunoblot analysis with the indicated antibodies of EGFR downstream pathway proteins. Statistically significant differences with *P*<0.05 were considered significant (**P<0.01; ***P<0.001)

The major EGFR downstream pathways include the Ras/Raf/MEK/Erk, PI3K/Akt, and STAT (signal transducer and activator of transcription ) pathways. As we have observed significant EGFR decrease with ganetespib treatment in a dose-dependent manner, we then were interested in revealing the effects of ganetespib on EGFR signaling cascades in GC cells. Therefore, phosphorylation levels of Erk1/2, JAK2 (Janus kinase 2), Src, and STAT3 were tested by western blots and were found to be substantially reduced with ganetespib treatment (Figure 5a). Marked changes of total Erk1/2, Akt, JAK2, Src, and PI3K were also observed. ELISA experiments of pSTAT3, pAkt, pJAK2 (phosphorylated JAK2), and p-Erk also confirmed the marked decrease of these proteins upon ganetespib treatment (Figure 5b). Importantly, pSTAT3, pAkt, pJAK2, and pErk were almost diminished upon HSP90 blockade in these experiments. These data suggest that blocking HSP90 could potentially interfere with EGFR cascades in GC cells.

To further confirm that blocking EGFR signaling cascades have an important role in the inhibitory effects of ganetespib on GC cells, siRNAs of *EGFR* were used. The knockdown efficiency of siEGFRs was studied and mRNA levels of *EGFR* were found to be significantly decreased by siEGFRs using siGL2 as a control (Figure 5c). In the cell-cycle experiments, MGC-803 cells transfected with either siEGFR had a significant increase of G2/M-phase cells, which was accompanied by a decrease in S-phase cells (Figure 5d). Apoptic cells in early and late apoptosis were significantly increased in cells transfected with either siEGFR (Figure 5e). siEGFRs also decreased protein levels of PI3K, pJAK2, pSTAT3, STAT3, pSrc, and pErk remarkably (Figure 5f). Taken together, these results suggest that response patterns of *EGFR* knockdown are similar to that observed in ganetespib treatment. These results indicated that EGFR signaling has an important role in ganetespib anticancer effects.

As mutated *EGFR*s often have an important role in tumorigenesis, it is necessary to examine whether there are mutations in EGFR proteins in the cell lines we studied. Therefore, we tried to sequence *EGFR* in MGC-803, SGC-7901, and MKN-28 cell lines. Four pairs of primers were designed to amplify four overlapped DNA fragments with cDNAs as templates (Supplementary Methods). Although there is one silent substitution (C720T) in *EGFR* of MGC-803 cells, it does not cause amino-acid changes in its protein (Supplementary Figures 2 and 3). Similarly, although there are two substitutions (G777A, T2133A) in *EGFR* of SGC-7901 cells, the amino acids of EGFR protein in SGC-7901 cells were not affected (Supplementary Figures 4 and 5). We were not able to obtain the mRNA sequence of *EGFR* in MKN-28 cell line, probably due to very low mRNA level of *EGFR* in MKN-28 cells (Supplementary Figure 6). *EGFR* gene in MKN-28 was previously fully sequenced by Catalogue of Somatic Mutations in Cancer (COSMIC),^30^ and no mutations were revealed. Besides, using genomic DNA of MKN-28, Gene Tech (Shanghai) company (Shanghai, China) performed *EGFR* mutation assays in exons 18, 19, 20, and 21, which are the hotspots of *EGFR* mutations in the current cancer research (Supplementary Figure 7), and no mutations in these regions were identified through pyrosequencing. Thus, we believe that *EGFR*s are wild-type in MGC-803, SGC-7901 and MKN-28 cell lines.

### Ganetespib in combination with cisplatin and radiation

**Figure 6:**
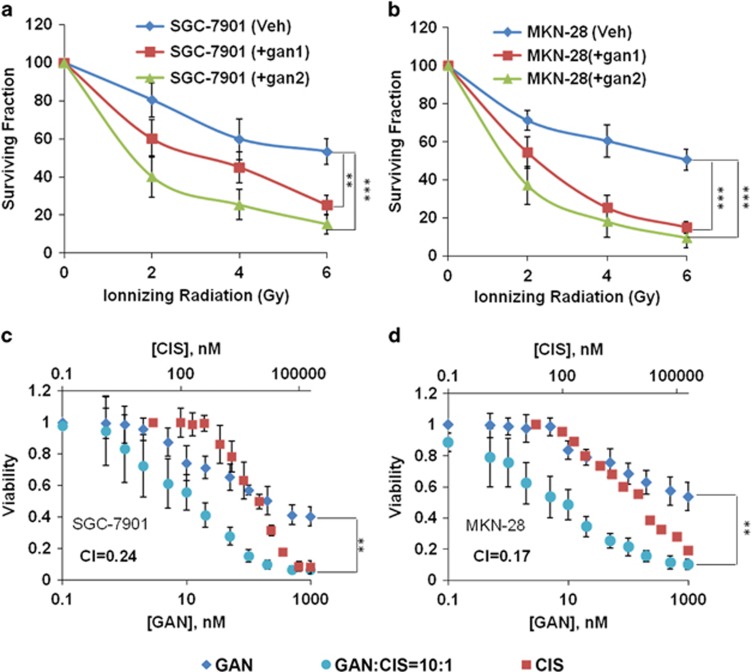
Ganetespib (GAN) treatment increased the radiosensitivity and chemosensitivity of GC cell lines. (**a**) Ganetespib treatment increased the radiosensitivity of SGC-7901 cells. The cells were treated with vehicle (blue), 75 nM (gan1, red) and 175 nM (gan2, green) ganetespib, and radiated at the indicated grade. (**b**) Ganetespib treatment increased the radiosensitivity of MKN-28 cells. The cells were treated with vehicle (blue), 92 nM (gan1, red) and 500 nM (gan2, green) ganetespib, and radiated at the indicated intensity. In (**a** and **b**), data indicate the mean surviving fraction calculated from three independent experiments (±S.E.). (**c** and **d**) Ganetespib treatment increased the cisplatin sensitivity in SGC-7901 (**c**) and MKN-28 (**d**) cells. The cells were treated with ganetespib (diamond), cisplatin (square) and ganetespib and cisplatin in combination (with a molar ratio of 10 : 1 (circles)) at the indicated concentration. Cells were exposed to the drugs for 72 h and cell viability was assessed by MTT Assay. Data indicate the mean percentage viability calculated from triplicate samples from three independent experiments (±S.E.). Statistically significant differences with *P*<0.05 were considered significant (**P<0.01; ***P<0.001)

Although the growth of poorly differentiated MGC-803 cells can be effectively inhibited by ganetespib at very low concentrations, the effects of ganetespib on moderately differentiated and highly differentiated cell lines, SGC-7901 and MKN-28, need to be explored further. SGC-7901 cells, which are neither sensitive to ganetespib treatment nor to radiation, are tried for the combined therapy of radiation and ganetespib (Figure 6a). The results have shown that ganetespib largely increased the sensitivity of SGC-7901 cells to radiation therapy. Similarly, ganetespib also increased sensitivity of MKN-28 cells to radiation (Figure 6b). More importantly, the results also showed that when combined with an appropriate dose of radiation (e.g., 4 Gy), ganetespib can kill >80% of the cancer cells at relatively low concentrations.

Cisplatin remains one of the most regularly used first-line cytotoxic agents in GC therapeutics. Thus, it was tested in combination with ganetespib for the treatment of SGC-7901 and MKN-28 cells, which were not very sensitive to ganetespib as a single agent. For this purpose, we conducted Chou–Talalay analysis,^31^ combining cisplatin with ganetespib in cultured SGC-7901 and MKN-28 cells. Ganetespib combined with cisplatin inhibited the growth of both SGC-7901 and MKN-28 cells much more significantly than either ganetespib or cisplatin administered individually (Figures 6c and d and data not shown). Notably, the resulting combination index (CI) values are <1, indicating that the combination of ganetespib was synergistic with cisplatin in both SGC-7901 and MKN-28 cells.

### Ganetespib inhibits tumor growth *in vivo*

**Figure 7:**
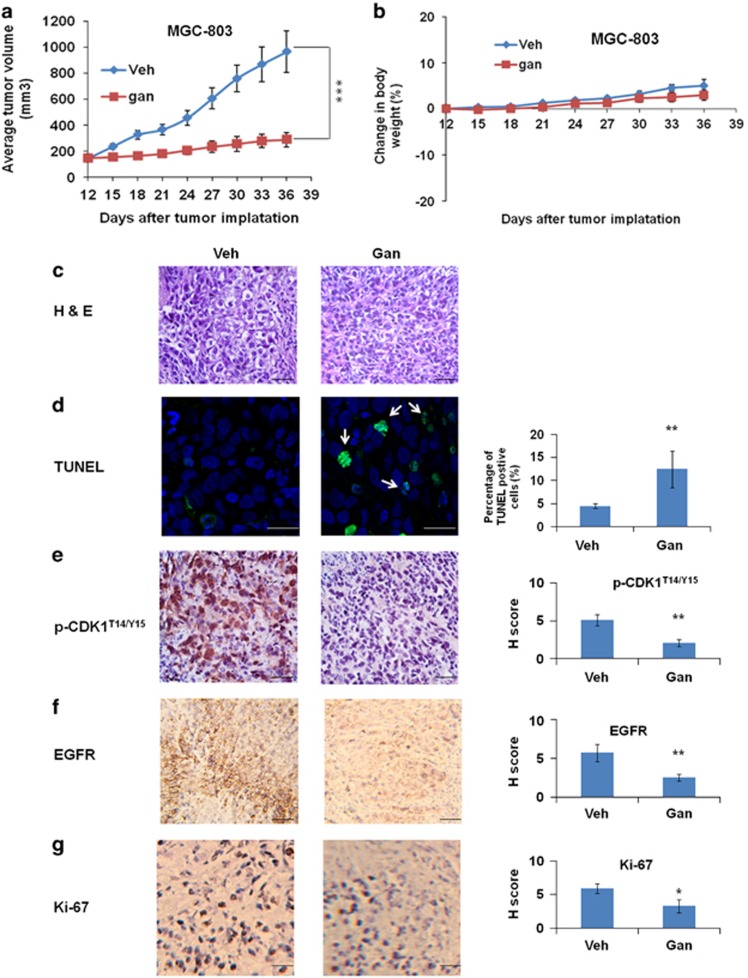
Ganetespib (gan) exhibits single-agent effects in a xenograft mouse model of GC. (**a**) Gan significantly inhibited tumor growth as a single agent in the xenograft mouse model of MGC-803 cells. Over a 3-week cycle, mice bearing established MGC-803 xenografts (*n*=6 per group) were intravenously dosed with 100 mg/kg ganetespib or vehicle once weekly. Data show average tumor volumes calculated at the indicated days and the error bars are the S.E.M. Differences among groups were compared by the Mann–Whitney test, with *P*<0.05 considered significant (***P<0.001). (**b**) Body weights of the xenograft mouse model of MGC-803 cells were measured every 3 days during the treatment. Mean values are plotted against vehicle controls and the error bars are the S.E.M. (**c**–**g**) Histology and immunohistochemistry of the xenograft mouse model of MGC-803 tumors treated with vehicle or ganetespib. Scale bar, 10 μm. (**c**) Hematoxylin and eosin (H&E)-stained tumor sections show a significantly larger number of signet ring cells in the vehicle-treated tissues than in ganetespib-treated ones. (**d**) Ganetespib significantly induced apoptosis in the xenograft mouse model. Confocal microscopic imaging of tumor sections showing green fluorescence TUNEL activity (white arrow) in combination with blue fluorescence DAPI (4',6-diamidino-2-phenylindole) in the nuclei. (**e–g**) Representative immunohistochemical (IHC) images analysis for phospho-CDK1^T14/Y15^ (**e**), EGFR (**f**), and Ki-67 (**g**) in the xenograft mouse model of MGC-803 tumors after vehicle or ganetespib treatment. Data are presented as the *H* score and the error bars are the S.E.M., considering both staining intensity and the percentage of positively staining cells. Statistically significant differences with *P*<0.05 were considered significant (*P<0.05; **P<0.01)

We next used xenograft mouse models of GC to evaluate *in vivo* efficacy of ganetespib as a single agent or in combination with cisplatin. In one experiment, the results showed that ganetespib treatment can significantly inhibit the growth of the tumors derived from MGC-803 cells (Figure 7a). The treatment was well tolerated, with no significant loss of body weights observed over the 3 weeks of dosing (Figure 7b). Hematoxylin/eosin (H&E) staining revealed that vehicle-treated tumor specimen had large and irregular nuclei and a large number of signet ring cells, and cells proliferated diffusely (Figure 7c). Generally, a significant number of signet ring cells are associated with a worse prognosis. On the other hand, tumor cells in the ganetespib-treated group were smaller in size and had very dark and dense nuclei (Figure 7c). With the earlier evidences that ganetespib induced apoptosis in MGC-803 cells, terminal deoxynucleotidyl transferase dUTP nick-end labeling (TUNEL) assay was carried out to assess whether it has similar effects *in vivo* (Figure 7d). Results have shown that about 12% of the ganetespib-treated cells became apoptotic as indicated by the co-occurrence of the TUNEL-positive green fluorescence and -positive blue nuclear fluorescence, which were significantly higher than TUNEL-positive cells detected in vehicle-treated tumors. These results supported the view that ganetespib may suppress the proliferation and induced apoptosis of MGC-803 cells. Immunohistochemical staining of phospho-CDK1^T14/Y15^, EGFR, and Ki-67, were further tested for cell-cycle arrest, correlation of ganetespib treatment with EGFR signaling, and proliferation. The results have shown significant reduced levels of phospho-CDK1^T14/Y15^ (Figure 7e), EGFR (Figure 7f), and Ki-67 (Figure 7g) in tumor tissues after ganetespib treatment.

**Figure 8:**
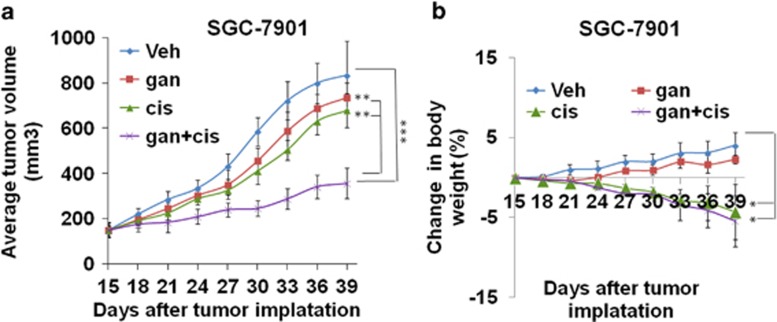
Ganetespib exhibits synergistic effects with cisplatin activity in a xenograft mouse model of GC. (**a**) Ganetespib exhibits significant synergistic effects with cisplatin in the xenograft mouse model of SGC-7901 cells. Tumors of SGC-7901 cells were treated with vehicle (veh, blue) or 100 mg/kg ganetespib (gan, red), 8 mg/kg cisplatin (cis, green), or 100 mg/kg ganetespib+8 mg/kg cisplatin (gan+cis, purple) starting from day 15. Data show average tumor volumes calculated at the indicated days and the error bars are the S.E.M. (**b**) Body weights of the xenograft mouse model of SGC-7901 cells were measured every 3 days during the treatment. Mean values are plotted against vehicle controls and the error bars are the S.E.M. Differences among groups were compared by the Mann–Whitney test, with *P*<0.05 considered significant (*P<0.05; **P<0.01; ***P<0.001)

In another experiment, the results have shown that neither ganetespib nor cisplatin can efficiently inhibit the growth of the tumors derived from SGC-7901 cells when they were applied individually at the experimental concentrations, although cisplatin treatment can mildly retard the growth of the tumors. Excitingly, when applied in combination with cisplatin, ganetespib can very efficiently inhibit the growth of the tumors. These results demonstrate that drug synergy between cisplatin and ganetespib can also be observed *in vivo* (Figure 8a). Cisplatin as a single agent and combination of cisplatin and ganetespib caused a mild (but significant) loss of body weights observed over the 3 weeks of dosing, but all treatments were tolerable (Figure 8b).

### EGFR sensitize additional poorly differentiated GC cell lines to ganetespib

**Figure 9:**
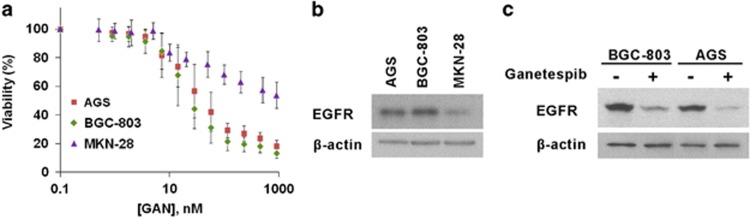
Ganetespib (Gan) treatment inhibits viabilities of poorly differentiated GC cell lines, and responsiveness of these cells lines to ganetespib treatment correlates well with their EGFR expression levels, with the well-differentiated cell line MKN-28 as the control. (**a**) Cells of GC cell lines BGC-803 (green diamonds), AGS (red squares), and MKN-28 (purple triangles) were treated with increasing concentrations of ganetespib for 72 h and cell viability was assessed by MTT assay at the indicated concentration. Data indicate the mean percentage viability calculated from triplicate samples from multiple independent experiments (*n*≥3) (±S.E.). (**b**) Representative immunoblots from cell lines BGC-803, AGS, and MKN-28 showing EGFR protein expression, with *β*-actin used as an internal control. (**c**) Representative immunoblots from cell lines BGC-803 and AGS showing EGFR protein expression after vehicle or ganetespib treatment (80 nM), with *β*-actin used as an internal control

In sum, the preceding data suggested considerable potential for inhibiting GC cell growth with ganetespib according to their expression profiles of EGFR. To gain additional insights into the correlation between responsiveness of GC cell lines and EGFR expression levels, a couple of additional poorly differentiated cell lines BGC-803 and AGS were used for assessment. As shown in Figure 9a, ganetespib treatment resulted in a dose-dependent inhibition of cell viability of these GC cell lines. The IC_50_ values of BGC-803 and AGS cells were 44.8 and 67.8 nM, respectively. The expression levels of EGFR in BGC-803 and AGS were much higher than that in MKN-28, and the IC_50_ values of ganetespib treatment in these two cell lines were much lower than that in MKN-28 (Figure 9b). In addition, the expression levels of EGFR decreased markedly after ganetespib treatment, which was also consistent with earlier observations in other GC cell lines (Figure 9c). Collectively, these findings again suggest that the protein levels of EGFR correlate very well with the responsiveness of GC cell lines to ganetespib treatment and have a critical role in sensitizing GC cell lines to ganetespib treatment.

## Discussion

In the past two decades, HSP90 inhibition has attracted much interest in cancer research field and there has been a considerable increase in the discovery of HSP90 inhibitors. Although first-generation HSP90 inhibitors have limited clinical uses because of their toxicity and issues related to solubility and formulation,^32, 33^ these inhibitors have shown promising anticancer activities and stimulated the discovery of the new-generation HSP90 inhibitors.^34, 35^ Thus far, several HSP90 inhibitors have shown antiproliferative and proapoptotic effects against GC *in vitro*, in animal models,^24, 36, 37, 38, 39, 40, 41^ and even in phase I clinical trials.^42^

In this study, we show that ganetespib significantly reduced the viability of MGC-803 cells, caused cell-cycle arrest, and induced cell apoptosis. In addition, ganetespib had similar inhibitory effects in cell lines SGC-7901 and MKN-28, except at higher concentrations of ganetespib. Besides, ganetespib significantly reduced tumor growth of MGC-803 cells in the absence of any observed drug-related toxicities. H&E staining, TUNEL assays, and immunohistochemistry results of phospho-CDK1^T14/Y15^ and Ki-67 again confirmed that ganetespib treatment inhibited proliferation of tumor cells, caused cell-cycle arrest, and induced cell apoptosis. Although MKN-28 and SGC-7901 cells are not very sensitive to ganetespib treatment, they can be effectively killed by the combination of ganetespib with radiation or cisplatin.

Mechanistically, ganetespib greatly affected EGFR signaling cascades by reducing EGFR proteins through increased EGFR trafficking and lowering the expression levels of proteins in downstream signaling pathways. These downstream cascade proteins, including JAK2, pSTAT3, EGFR, and ERK, are linked to GC pathogenesis. *EGFR* knockdown through siRNAs caused similar response patterns as ganetespib treatment, such as cell-cycle arrest, apoptosis, and decreases of downstream protein levels. Immunohistochemistry results of EGFR in ganetespib-treated tumor tissues confirmed that the protein levels of EGFR decreased markedly *in vivo*. Although we cannot absolutely rule out the possibility that other client proteins of HSP90 may also contribute to the inhibitory effects of HSP90, all these findings strongly suggest that EGFR signaling has an important role in sensitizing GC cell lines to ganetespib.

Furthermore, our results have shown that EGFR expression levels in cell lines directly correlate with their sensitivity to ganetespib treatment. The most attractive point in our study is that the most aggressive and poorly differentiated GC cell lines, MGC-803, with the highest EGFR expression, is the most vulnerable to ganetespib treatment. This point was further confirmed by testing two additional poorly differentiated GC cell lines AGS and BGC-803. They both have much higher EGFR expression levels than highly differentiated cell line MKN-28 and responded to ganetespib much better than MKN-28. These results may provide a new potent treatment option for GC patients with high EGFR expression.

Currently, recurrent GC patients with resistance to chemotherapy drugs are often lack of effective treatment options. Ganetespib have worked well in inhibiting cisplatin-resistant GC cell lines *in vitro* and in mouse models. Such encouraging results have suggested potential benefits for recurrent GC patients to try treatment using ganetespib.

## Materials and Methods

### Cell culture

Human GC poorly differentiated cell lines MGC-803, AGS, and BGC-803, moderately differentiated cell line SGC-7901, highly differentiated MKN-28, and normal gastric mucosal epithelial cell line GES-1 were maintained in DMEM or RPMI1640 (Life Technologies (Invitrogen), Grand Island, NY, USA), with 10% FBS (Thermo Scientific (Hyclone, Logan, UT, USA)), penicillin/streptomycin (100 U/ml and 100 *μ*g/ml, respectively; Life Technologies (Invitrogen)).

### Antibodies and chemical reagents used

The antibodies used and commercial sources are as follows: cyclin B1, CHK1, EGFR, AKT, pAKT^S473^, JAK2, pJAK2^Y1007/1008^, STAT3, pSTAT3^Y705^, c-Myc, Met. IGF-1 R, cleaved PARP^D214^, cleaved caspase-3^D175^, cleaved caspase-7^D198^, cleaved caspase-9^D330^, Src, pSrc ^Y418^, PI3K (p110*β*), Erk, pErk ^T202/Y204^, S6, pS6^S235/236^ (Cell Signaling, Danvers, MA, USA); CDK1, Ki-67 (Abcam, Cambridge, MA, USA); and pCDK1(Thr14/Tyr15), *β*-actin (Sigma-Aldrich, St. Louis, MO, USA); p-EGFR^Y1173^ (Life Technologies (Invitrogen)). The drugs, ganetespib, dasatinib, alisertib, everolimus, and cisplatin, were purchased from Selleck Chemicals Inc. (Houston, TX, USA). The siRNAs siGL2 (targeting firefly luciferase GL2), siEGFR1, and siEGFR2 (targeting human EGFR) were synthesized by Shanghai GenePharma Co. (Shanghai, China) and transfected with Lipofectamine 2000 (Life Technologies, Grand Island, NY, USA). The siRNAs used are as follows: siGL2, (F) 5′- UCGAAGUAUUCCGCGUACG-3′ and (R) 5′-CGUACGCGGAAUACUUCGA-3′ siEGFR1, (F) 5′- GGAGAUAAGUGAUGGAGAUTT-3′ and (R) 5′- AUCUCCAUCACUUAUCUCCTT-3′ siEGFR2, (F) 5′-CCCAGAAGGUGAGAAAGUUTT-3′ and (R) 5′- AACUUUCUCACCUUCUGGGTT-3′.

### Cell viability

Cells were plated in 96-well plates (3000 cells/well). After the cells were attached to the bottom, different drugs (ganetespib, dasatinib, alisertib, cisplatin, or everolimus) were added into the wells to meet the desirable concentrations. After 72 h, cell viability was determined using MTT (3-[4]-2,5- diphenyltetrazolium bromide thiazolyl blue) reagents (KeyGen Biotech Inc., Nanjing, China) according to the manufacturer's instructions.

### Apoptosis and cell-cycle assays

Apoptosis was evaluated by Annexin V-FITC/PI staining reagent (Yeasen Biotech, Shanghai, China) in ganetespib-treated or siRNA-transfected cells for 72 h. Briefly, cells in the medium were spinned down and kept in a centrifuge tube. The attached cells were trypsinized without EDTA and harvested from tissue culture plates, combined with cells in the medium, and then centrifuged at 300 × *g* for 5 min at 4 °C. Cells were washed two times with prechilled PBS and suspended in 100 *μ*l of binding buffer. Each sample was added with 5 *μ*l of Annexin V-FITC and 5 *μ*l of PI staining reagents. The samples were stained for 10 min at room temperature in the dark and then 400 *μ*l of binding buffer was added to each sample. The samples were analyzed using Gallios Flow Cytometer (Beckman Coulter, Brea, CA, USA) available in the Analytical and Testing Center at Jiangsu University (Jiangsu, China). Annexin V (+)/PI (−) cells were identified in the early stages of apoptosis and Annexin V (+)/PI (+) cells were identified in the late stages. For cell-cycle analysis, cells were seeded at 9 × 10^4^ cells/well in a 6-well plate and were exposed to ganetespib or siRNAs. After 24 h, cells were harvested and stained with propidium iodide (Sigma-Aldrich), and analyzed on the Gallios Flow Cytometer according to the manufacturer's instructions. Flow cytometry data were analyzed using the FlowJo software (Ashland, OR, USA).

### Drug synergy testing

Ganetespib and cisplatin were tested individually or in combination in GC cell lines, including SGC-7901 and MKN-28. Cells were plated at 3000 cells per well in 96-well plates. After 24 h of incubation, cells were treated with serial dilutions of combinations of two drugs at a constant molar ratio as indicated. After 72 h of incubation, cell viability was measured with MTT reagents. CI values was established by the Chou–Talalay method^31, 43, 44^ calculated using the CompuSyn software package (ComboSyn Inc., Paramus, NJ, USA).

### Immunoblot, ELISA assays, and analysis

Cells were lysed in Mammalian Protein Extraction Reagent (MPER) (Thermo Scientific, Rockford, IL, USA). MPER buffer was supplemented with Halt™ Protease and Phosphatase Inhibitor Cocktail (Thermo Scientific, Rockford, IL, USA), and protein concentrations were determined using the BCA (bicinchoninic acid) assay (Thermo Scientific). Proteins were resolved on 8–12% SDS-PAGE gels and transferred to the PVDF membrane (EMD Millipore, Billerica, MA, USA). Membranes were blocked in non-fat dry milk, incubated overnight at 4 °C in a primary antibody, followed by a horseradish peroxidase-conjugated secondary antibody (GE Healthcare, Pittsburgh, PA, USA), and signals were detected with SuperSignal West Pico Chemiluminescent Substrate (Thermo Scientific). Expression levels of indicated proteins present in protein lysates after ganetespib treatment were assayed by ELISA assays (reagents from Roche Diagnostics (Indianapolis, IN )) using a Biotek Synergy HT reader (Biotek, Winooski, VT, USA) according to the manufacturer's instructions.

### Combination effects of radiation and ganetespib on GC cells

SGC-7901 and MKN-28 cells were seeded into 60 mm dishes (2 × 10^4^ cells/dish). After 24 h, cells were then radiated with 0, 2, 4, and 6 Gy of radiation at Nanjing Radiation Center (Nanjing, China). After the cells were allowed to recover for 12 h, the radiated cells were treated with vehicle or ganetespib at two different doses and then cultured for additional 3 days. Then, 1% of the cells in each dish were plated in a new dish and cultured for additional 14 days. The colony numbers of each group were counted and normalized against their control without ionization radiation. Three independent experiments were performed for each condition and standard deviations were determined.

### Immunofluorescence staining

MGC-803 cells were seeded on glass slides in the presence or absence of ganetespib. After 24 h of ganetespib treatment, cells were either directly fixed with freshly prepared 4% paraformaldehyde (Electron Microscopy Sciences) or stimulated with 100 ng/ml of EGF-Alexa 488 for 15 min and then fixed in 4% paraformaldehyde/PBS at ambient temperature for 10 min. Fixation was followed by two washes in PBS and stored at 4 °C or labeled immediately. Cells were incubated in PBS with 5% normal bovine serum and 0.3% Triton X-100, followed by 1 h incubation with primary antibodies diluted 1 : 100 in 1% BSA/0.3% Triton X-100/PBS and incubation with appropriate secondary antibodies conjugated with Alexa Fluor 488 or Alexa Fluor 568 (Invitrogen) as indicated. All immunofluorescence imaging experiments were repeated at least three times. In multiple spots of each slide, 10 or more fully adherent and viable (judged by labeled epidermal growth factor (EGF) internalization) cells were analyzed for each condition. Optical sections through the middle of the cells were acquired on a Nikon C1 Spectral Confocal microscope (Nikon Instruments Co., Melville, NY, USA) with × 60 oil objective. Analysis for marker colocalization was carried out with Image-Pro software using the built-in colocalization tool. The threshold of background was applied uniformly to single-channel images per individual cells so that the intensity of EEA1 did not include the diffuse background staining. The extent of colocalization was calculated as the spatial overlap between two channels, which is expressed as the ratio of the integrated EGF fluorescence within the indicated endosomal compartment marker EEA1 to the total EGF intensity.

### Tumor killing activity

All procedures involving mice were approved by the Jiangsu University Institutional Animal Care and Use Committee. All mice were provided with sterilized food and water and housed in a barrier facility under a 12-h light–dark cycle. Xenograft tumor-bearing Nu/Nu nude mice were created according to the standard protocol. In one experiment, the 6-week-old Nu/Nu nude mice were injected subcutaneously in the middle of the left flank with 100 *μ*l of a single-cell suspension containing 2 × 10^6^ MGC-803 cells. Tumors were measured every 3 days with vernier calipers, and volumes were calculated according to the formula: volume=0.52 × *W*^2^ × *L*, where *W* and *L* represent the width and length, respectively. Drug treatment started when the tumor size reached ~100–150 mm^3^ (12 days after initial implantation), and the mice were randomized into two groups (6 mice per group). Ganetespib, 100 mg/kg formulated in 10/18 DRD (10% DMSO, 18% Cremophor RH 40, 3.6% dextrose, and 68.4% water) or 10/18 DRD (vehicle), was administered once a week by tail vein injection for 3 weeks. At the end of the observation (36 days after the initial implantation and 24 days after treatment), mice were killed and autopsied. Tumors were removed and weighed. Differences among groups were compared by the Mann–Whitney test with *P*<0.05 considered significant.

Similarly, in another experiment, the 6-week-old Nu/Nu nude mice were injected subcutaneously in the middle of the left flank with 100 *μ*l of a single-cell suspension containing 2 × 10^6^ SGC-7901 cells. After tumors formed (about 150 mm^3^), mice were randomly divided into four groups. Four groups (6 mice/group) were: (i) vehicle (negative control); (ii) ganetespib; (iii) cisplatin; and (iv) ganetespib plus cisplatin. Ganetespib, 100 mg/kg formulated in 10/18 DRD or 10/18 DRD (vehicle), was administered once weekly by tail vein injection. Cisplatin was prepared freshly on the day of administration in normal saline at a concentration of 1 mg/ml. Cisplatin was injected intraperitoneally at a dose of 8 mg/kg once a week. Mice with SGC-7901 xenografts were treated for 3 weeks. Tumor volume was monitored once every 3 days after injections. Differences among groups were compared by the Mann–Whitney test with *P*<0.05 considered significant.

### Tissue preparation, histology, and immunohistochemistry

Individual portions of tumors were fixed in 10% (v/v) neutral-buffered formalin and paraffin-embedded and cut. Tissues were sectioned at 5 *μ*m and sections were deparaffinized in xylenes and hydrated in a graded series of alcohols. Then, sections were either hematoxylin and eosin (KeyGen Biotech) stained for morphological evaluation or subjected to TUNEL assays (Vazyme Biotech Co., Nanjing, China) or antigen retrieval for immunohistochemistry studies. Antigen retrieval was conducted by heating slides in EDTA buffer (pH 9.0) for 20 min or subjected to pepsin digestion at 37 °C for 15 min. Endogenous peroxidase was blocked by preincubation with 0.3% hydrogen peroxide for 15 min. The nonspecific antigens were blocked by 10% goat serum in PBS, and sections were incubated with the following primary antibodies at the indicated dilutions: Ki-67 (1 : 100), EGFR (1 : 50), and pCDK1^T14/Y15^ (1 : 50) at room temperature for 1 h in a humidified chamber. Sections were rinsed with PBS and exposed to appropriate species-specific secondary antibodies for 30 min. Finally, each section was exposed to 3,3′-diaminobenzidine (DAB) solution (KeyGen Biotech) for 3–5 min after they were rinsed with PBS. Immunostained sections were lightly counterstained with hematoxylin (KeyGen Biotech), dehydrated through a series of alcohols and xylenes, and coverslipped.

### Evaluation of immunohistochemical staining

Immunoreactivity was evaluated independently by two researchers who were blinded to tumor origins. The evaluation was based on the staining intensity and extent of staining. Staining intensity was scored as 0 (negative), 1 (weak), 2 (moderate), and 3 (strong). Staining extent was scored as 0 (0%), 1 (1–25%), 2 (26–50%), 3 (51–75%), and 4 (76–100%), depending on the percentage of positive-stained cells. The sum of the staining intensity and the staining extent scores was used as the final staining score. In the event of a discrepancy in scoring, the slides were re-examined by both pathologists.

### Quantitative RT-PCR

To evaluate the expression of EGFR, each of the cell lines was grown to 70% confluence in complete medium, and then total RNA was extracted with RNeasy Mini Kit (Qiagen, Valencia, CA, USA). Quantitative RT-PCRs were performed with TaqMan probes and primers in an ABI PRISM 7700 detection system (Applied Biosystems, Foster City, CA, USA). The results were analyzed with the comparative Ct method to establish relative expression curves, and glyceraldehyde 3-phosphate dehydrogenase (GAPDH) was used as an internal control. The probes used are as follows: EGFR, (F) 5′-CGCAAAGTGTGTAACGGAATAGGTA-3′ and (R) 5′-CCAGAGGAGGAGTATGTGTGAAGGA-3′ GAPDH, (F) 5′-GAAGGTGAAGGTCGGAGTC-3′ and (R) 5′-GAAGATGGTGATGGGATTTC-3′.

### Statistical analysis

Statistically significant differences between different groups were determined by two-way ANOVA, followed by Bonferroni post-tests, with *P*<0.05 considered significant (*P<0.05; **P<0.01; ***P<0.001) using GraphPad Prism version 5.00 (GraphPad, San Diego, CA, USA), unless otherwise specified.

## Abbreviations

GC: gastric cancer
HSP90: heat-shock protein 90
(phospho-, p) EGFR: phosphorylated epidermal growth factor receptor
H&E: hematoxylin/eosin
TUNEL: terminal deoxynucleotidyl transferase dUTP nick-end labeling
(phospho-, p) CDK1: phosphorylated cyclin-dependent kinase 1
(phospho-, p) mTOR: (phosphorylated) mammalian target of rapamycin
PI3K: phosphoinositide 3-kinase
siRNA: small interfering RNA
HIF-1: hypoxia-inducible factor-1
VEGFR: vascular endothelial growth factor receptor
ATP: adenosine triphosphate
CHK1: checkpoint kinase 1
(phospho-, p) JAK2: phosphorylated Janus kinase 2
(phospho-, p) STAT3: (phosphorylated) signal transducer and activator of transcription 3
PARP: poly ADP ribose polymerase
Erk: extracellular-signal-regulated kinase
MTT: 3-(4, 5-dimethylthiazol-2-yl)-2,5-diphenyl tetrazolium bromide or Thiazolyl blue
CI: combination index
EGF: epidermal growth factor
EEA: early endosome antigen 1
GAPDH: glyceraldehyde 3-phosphate dehydrogenase
IC50: half-maximal inhibitory concentration
IGF-1R: insulin-like growth factor-1 receptor
