# Supplementary material for: Targeting heat-shock protein 90 with ganetespib for molecularly targeted therapy of gastric cancer
Source: Cell Death Dis. 2015 Jan 15;6(1):e1595–. doi: 10.1038/cddis.2014.555 (PMC4669753; doi:10.1038/cddis.2014.555)
Supplement: Supplementary Information [file cddis2014555x1.doc]

**Table of Contents:**

**Supplemental Methods – page 2**

**Supplemental Table 1 – page 3**

**Supplemental Figure 1– page 4-5**

**Supplemental Figure 2 –page 6**

**Supplemental Figure 3 –page 7**

**Supplemental Figure 4 –page 8**

**Supplemental Figure 5 –page 9**

**Supplemental Figure 6 –page 10**

**Supplemental Figure 7 –page 11**

**Supplemental Methods.**

**EGFR sequencing**

MGC-803, SGC-7901 and MKN-28 cells were subjected to the sequencing. Cells were harvested and washed with PBS twice. Total RNA was extracted by using TRIzol Reagent (Invitrogen) following the manufacturer’s instruction. Complementary DNA (cDNA) was generated through reverse transcription by using iScript™ cDNA Synthesis Kit (Life Technologies). Four overlapped DNA fragments were amplified by using the primers in Table 1 and the cDNAs as the templates. Pfu DNA polymerase (Thermo Fisher) was used in the PCR reactions. The thermal cycles are as the following: 95℃ for 5 minutes; and then 95℃ for 30 seconds, 60℃ for 30 seconds and 72℃ for 2 minutes, 30 cycles; 72℃ for 10 minutes; 4℃. The DNA fragments were sequenced by BGI Life Tech Co., Ltd. Each fragment was successfully sequenced at least three times to ensure the accuracy of the sequencing. The obtained DNA sequences were analyzed by using DNAStar software and compared to the published mRNA sequence of EGFR (NM_005228.3). The obtained mRNA sequences of EGFR of MGC-803 and SGC-7901 cell lines were translated and compared with the published protein sequence of wild-type EGFR (NP_005219.2).

**Supplemental Table 1. Primers for sequencing mRNAs of EGFR.**

| Primer Pairs | Up-stream (5’-3’) | Down-stream (5’-3’) |
| --- | --- | --- |
| EGFR-1 | tgactccgtccagtattgatc | cgtagcatttatggagagtgag |
| EGFR-2 | acagctatgagatggaggaagac | acaagctccctctcctgcagca |
| EGFR-3 | tccatcgccactgggatggt | tgctgtgggatgaggtact |
| EGFR-4 | cagggggatgaaagaatgca | caaaaccagtctgtgggtct |

**
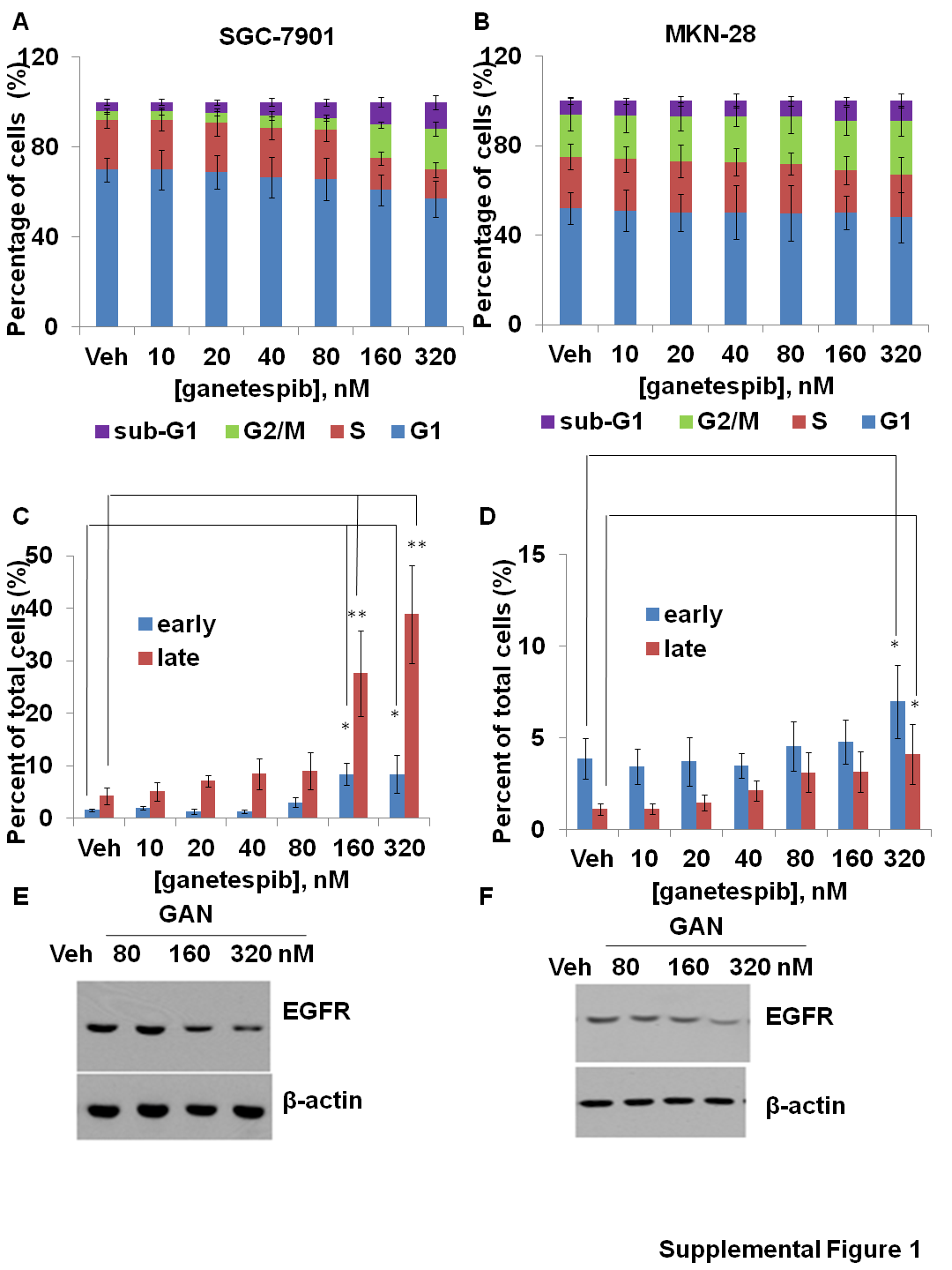
**

**Supplemental Figure 1. Ganetespib treatment induces G2/M cell-cycle arrest, apoptosis and caused EGFR decrease in gastric cancer cells. A-B**, data shown are the mean values (±SE) of each cell cycle phase from three independent experiments. SGC-7901 (**A**) and MKN-28 (**B**) cells were treated with 0, 10, 20, 40, 80, 160, or 320 nmol/L ganetespib for 24 hours, stained with propidium iodide and analyzed for cell-cycle distribution. **C-D**, SGC-7901 (**C**) and MKN-28 (**D**) cells were treated with 0, 10, 20, 40, 80, 160, or 320 nmol/L ganetespib for 72 hours, stained with Annexin V-FITC/propidium iodide and analyzed for cell apoptosis distribution. The cells were analyzed for the presence of Annexin V (+)/PI (-) (early apoptosis) and Annexin V (+)/PI (+) (late apoptosis). Data shown are the mean values (±SE) from three independent experiments. E-F, SGC-7901 (**E**) and MKN-28 (**F**) cells were treated with 0, 80, 160, or 320 nmol/L ganetespib for 24 hours, and protein lysates were subjected to immunoblot analysis with the indicated antibodies.

**Supplemental Figure 2.**

**
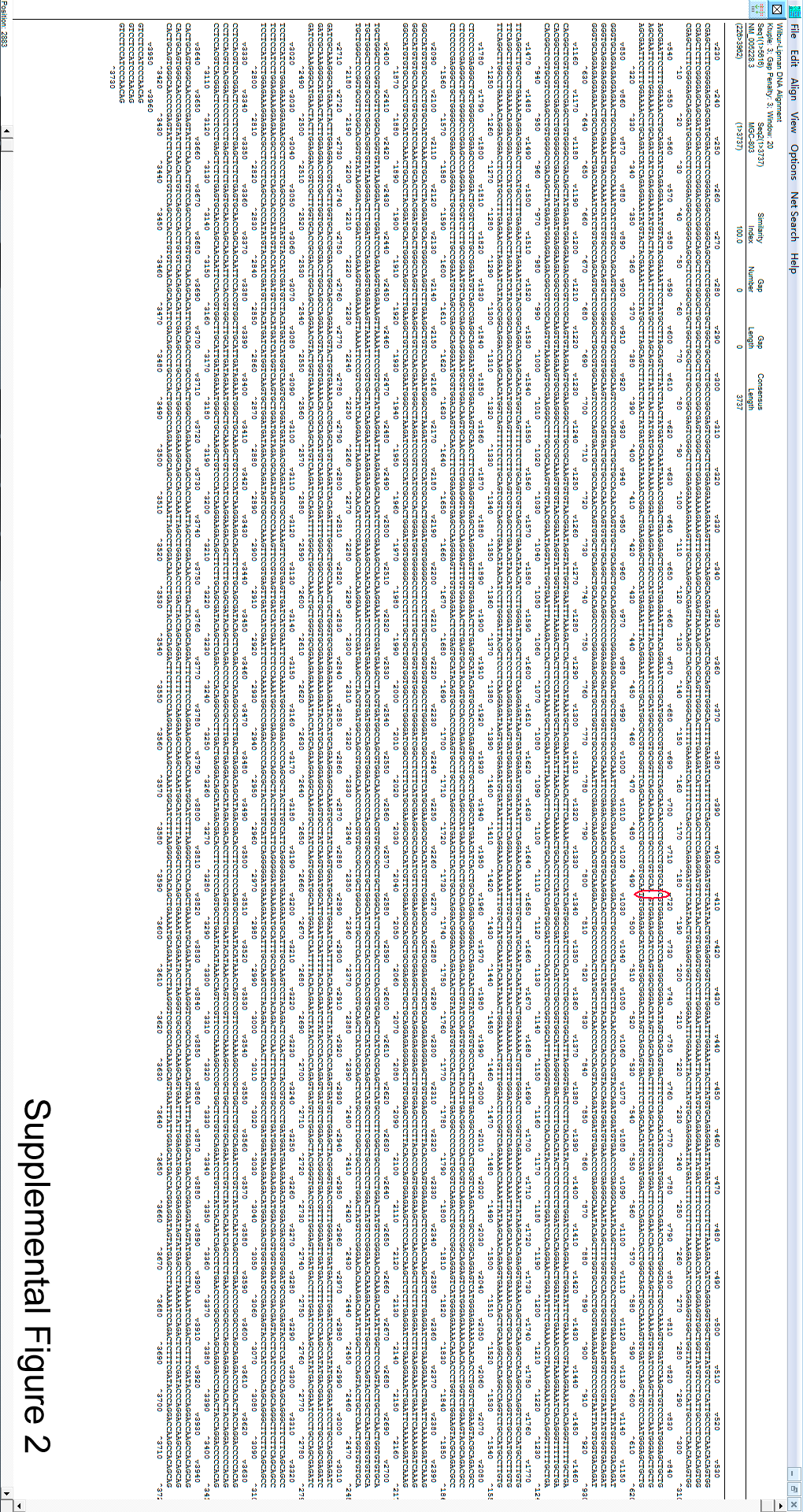
**

**Supplemental Figure 2. Sequencing result of EGFR mRNA in MGC-803 cell line.** One substitution (C720T) in EGFR of MGC-803 was identified.


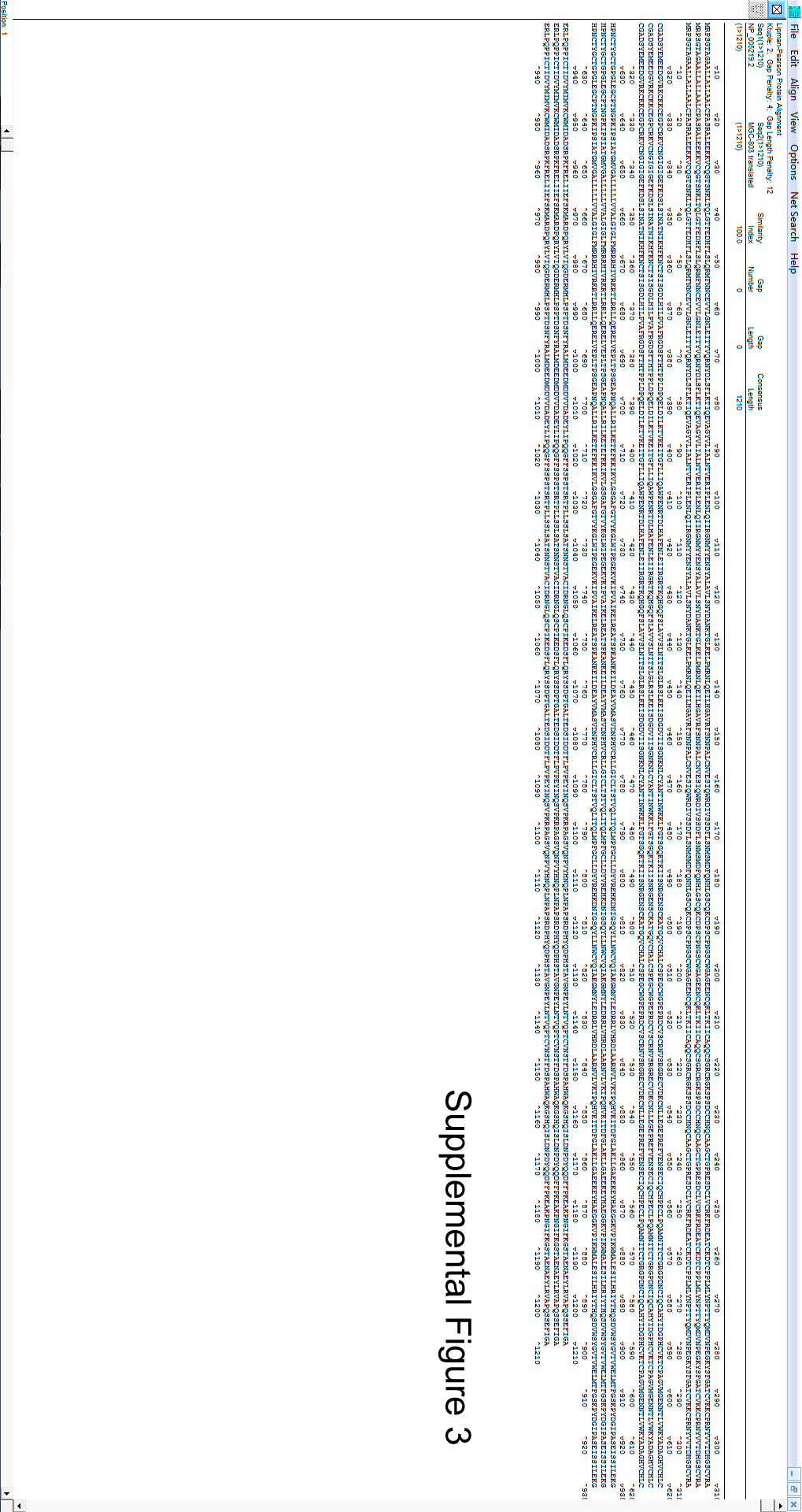
**Supplemental Figure 3.**

**Supplemental Figure 3. Segueing result of EGFR protein in MGC-803 cell line.** No amino acid changes were identified in EGFR of MGC-803 were identified.

**Supplemental Figure 4.**


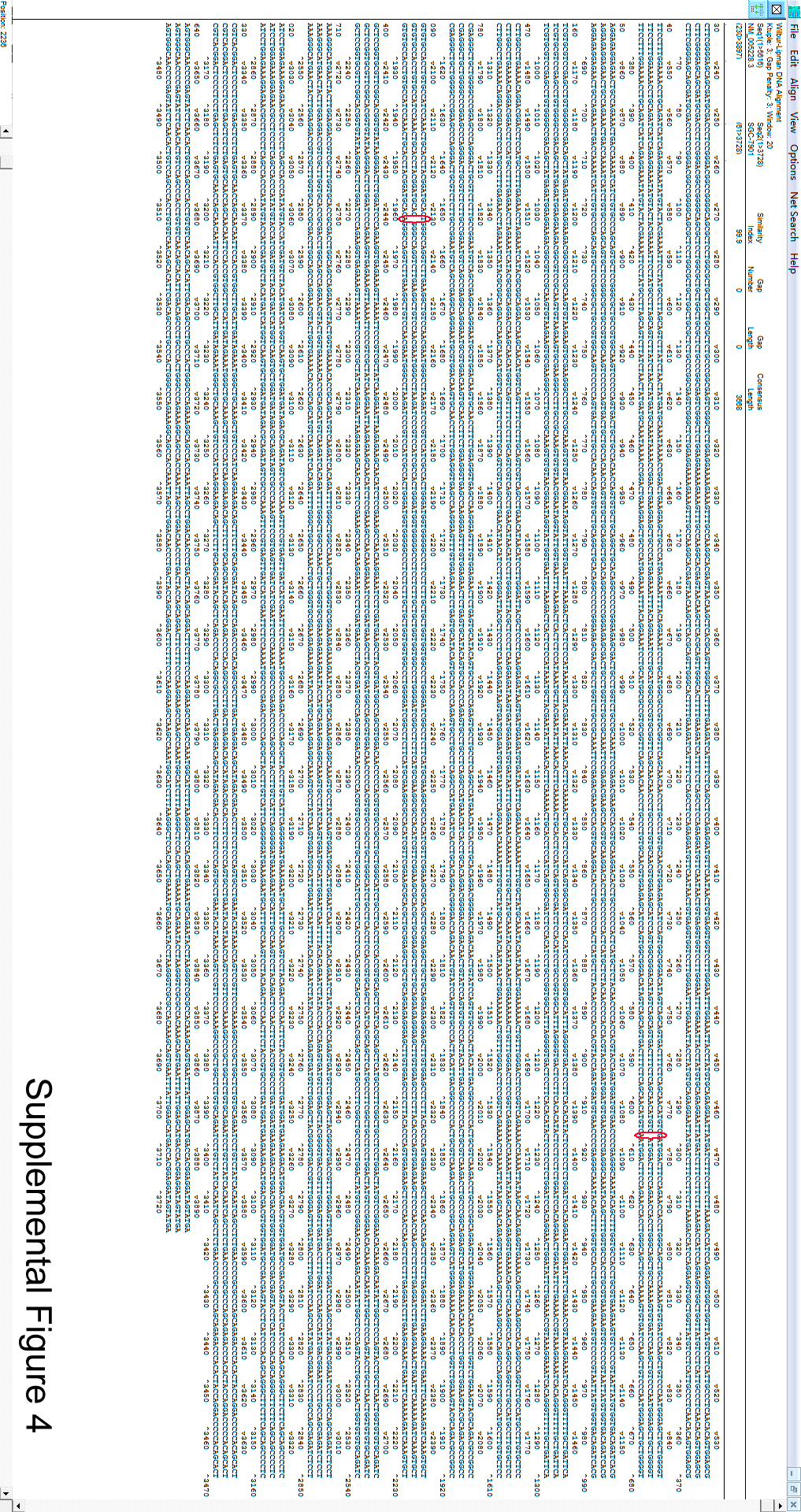
**Supplemental Figure 4. Segueing result of EGFR mRNA in SGC-7901 cell line.** Two substitutions (G777A, T2133A) in EGFR of SGC-7901 cells were identified.


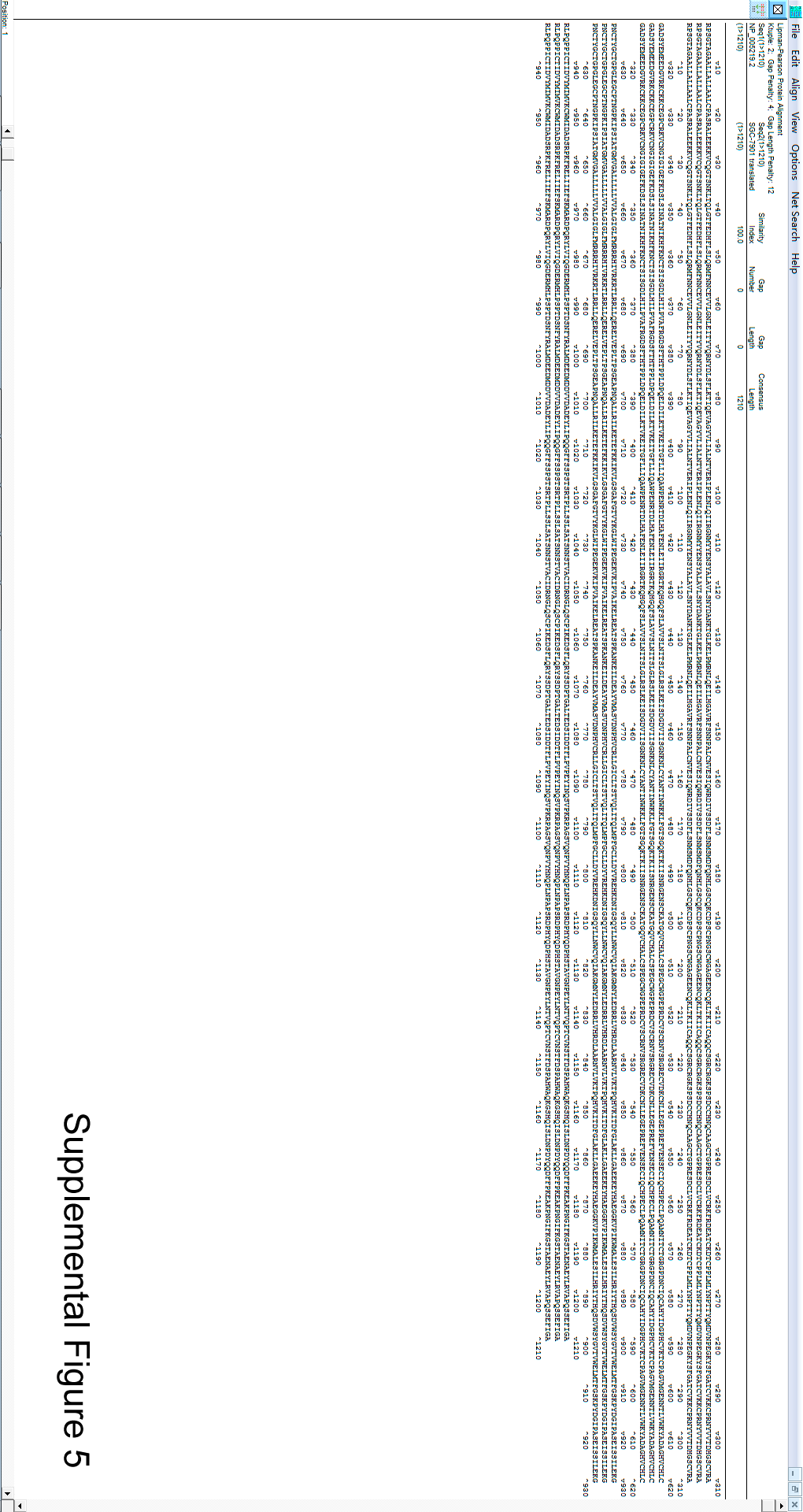
**Supplemental Figure 5.**

**Supplemental Figure 5. Sequencing result of EGFR protein in SGC-7901 cell line.** No amino acid changes were identified in EGFR of SGC-7901 were identified.

**
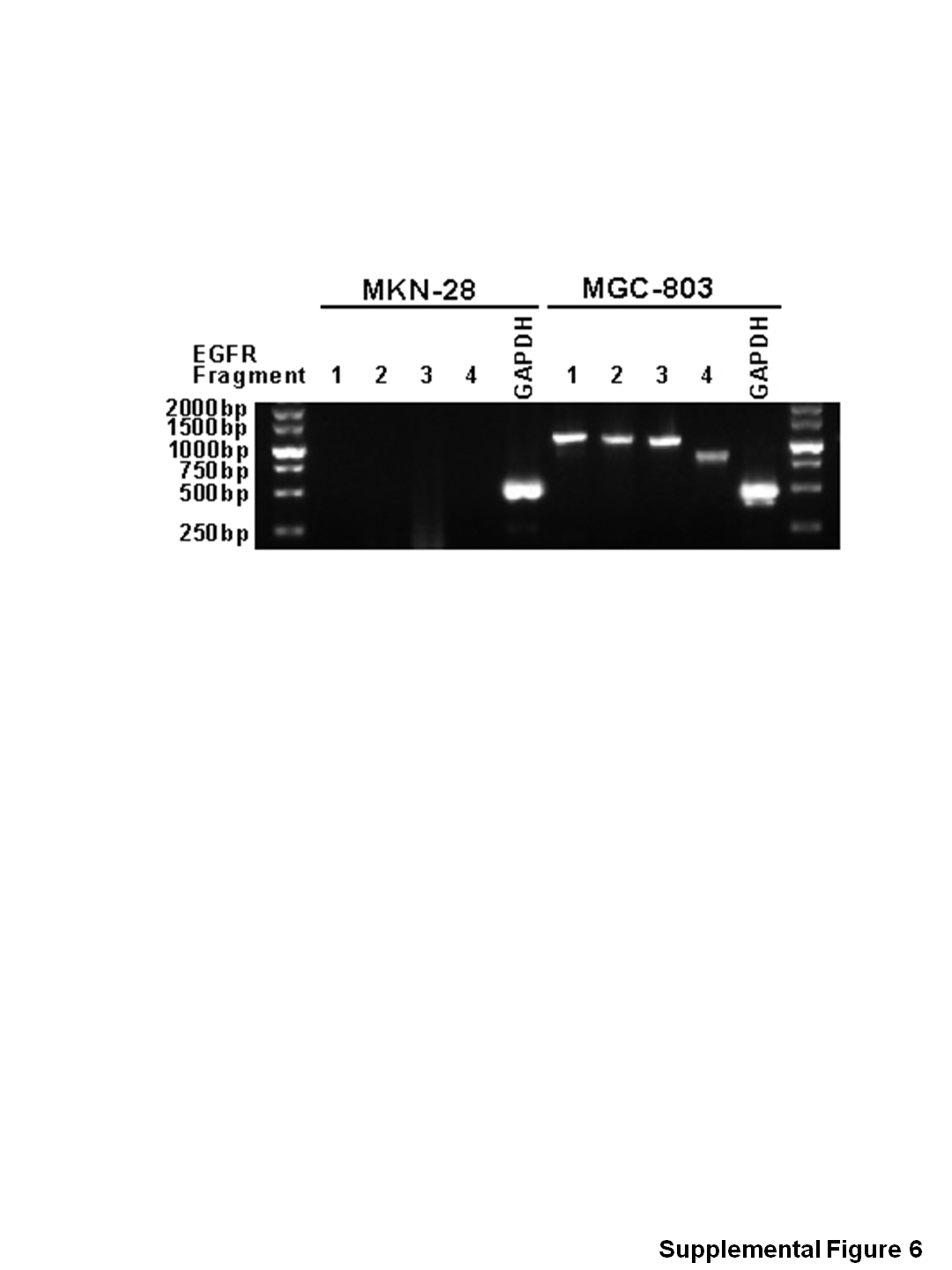
**

**Supplemental Figure 6. Reverse Transcription PCR (RT-PCR) to detect EGFR mRNA in MKN-28 and MGC-803 cell lines.** Four DNA fragments were amplified from EGFR mRNA of MGC-803 cells. No DNA fragments have been amplified from mRNA of MKN-28 cell line, with GAPDH  used as an internal control. The primers for GAPDH in this experiment are: 5’-CAAGGTCATCCATGACAACTTTG-3’and

5’-GTCCACCACCCTGTTGCTGTAG-3’.

**
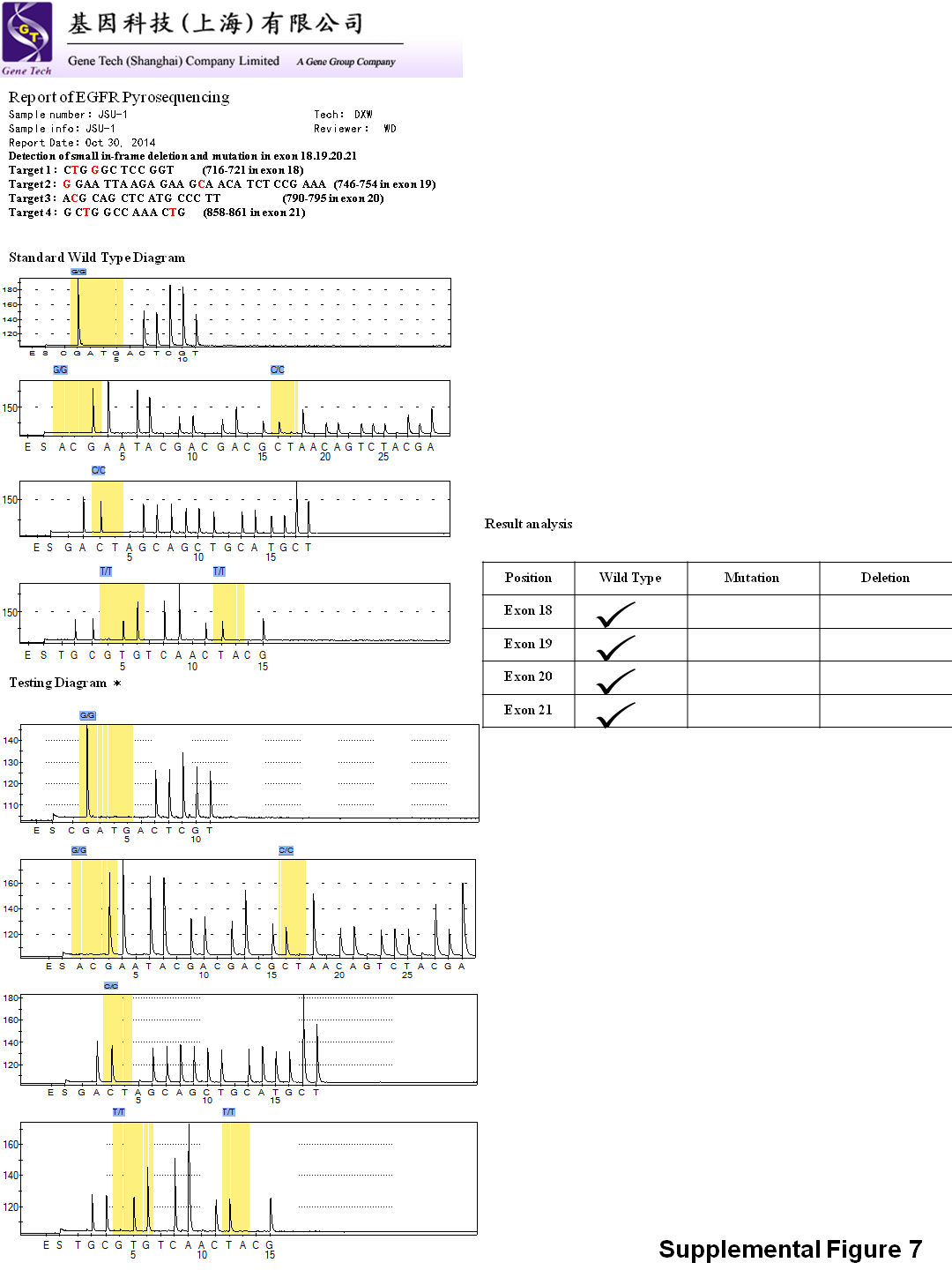
**

**Supplemental Figure 7. EGFR mutation hotspot assay report from Gene Tech (Shanghai) Co. shows no mutations identified in the regions of 716-721 in exon 18, 746-754 in exon 19, 790-795 in exon 20, and 858-861 in exon 21 in EGFR gene of MKN-28 cell line.** DNA genomes in MKN-28 were used for the assays and were extracted and treated following company’s instructions.
